# Could interventions on physical activity mitigate genomic liability for obesity? Applying the health disparity framework in genetically informed studies

**DOI:** 10.1007/s10654-023-00980-y

**Published:** 2023-03-11

**Authors:** Moritz Herle, Andrew Pickles, Oliver Pain, Russell Viner, Jean-Baptiste Pingault, Bianca L. De Stavola

**Affiliations:** 1 Department of Biostatistics and Health Informatics, Institute of Psychiatry, Psychology & Neuroscience, Kings College London, London, UK; 2 Social, Genetic and Developmental Psychiatry Centre, Institute of Psychiatry, Psychology & Neuroscience, Kings College London, 16 De Crespigny Park, Denmark Hill, London, SE5 8AF UK; 3 Maurice Wohl Clinical Neuroscience Institute, Department of Basic and Clinical Neuroscience, Institute of Psychiatry, Psychology & Neuroscience, King’s College London, London, UK; 4 Population, Policy and Practice Research and Teaching Department, UCL Great Ormond Street Institute of Child Health, University College London, London, UK; 5 Division of Psychology and Language Sciences, Department of Clinical, Educational and Health Psychology, University College London, London, UK

**Keywords:** ALSPAC, Polygenic score, Longitudinal study, BMI, Causal inference, Mediation analysis, MCS

## Abstract

Polygenic scores (PGS) are now commonly available in longitudinal cohort studies, leading to their integration into epidemiological research. In this work, our aim is to explore how polygenic scores can be used as exposures in causal inference-based methods, specifically mediation analyses. We propose to estimate the extent to which the association of a polygenic score indexing genetic liability to an outcome could be mitigated by a potential intervention on a mediator. To do this this, we use the interventional disparity measure approach, which allows us to compare the adjusted total effect of an exposure on an outcome, with the association that would remain had we intervened on a potentially modifiable mediator. As an example, we analyse data from two UK cohorts, the Millennium Cohort Study (MCS, N = 2575) and the Avon Longitudinal Study of Parents and Children (ALSPAC, N = 3347). In both, the exposure is genetic liability for obesity (indicated by a PGS for BMI), the outcome is late childhood/early adolescent BMI, and the mediator and potential intervention target is physical activity, measured between exposure and outcome. Our results suggest that a potential intervention on child physical activity can mitigate some of the genetic liability for childhood obesity. We propose that including PGSs in a health disparity measure approach, and causal inference-based methods more broadly, is a valuable addition to the study of gene-environment interplay in complex health outcomes.

**Supplementary Information:**

The online version contains supplementary material available at 10.1007/s10654-023-00980-y.

## Introduction

Leveraging observational data to estimate causal links between exposures and health outcomes, when randomised control trials are not possible, has been one of the main aims in epidemiology. To that end, methods have been developed to derive causal inferences from observational data, aiming to handle bias introduced from confounding and reverse causation by mimicking features of randomised control trials. This is done by using tools such as instrumental variables, propensity scores matching or reweighting [1]. These methods assume that the cause of interest can be intervened on and results into a specific and tightly defined causal effect on the outcome [1]. However, there are many other instances where it is not possible to define a potential intervention, as exposures of interest are non-manipulatable and have non-specific wide-reaching consequences (e.g. ethnicity, sex or socio-economic position). In this situation, it has been proposed to shift attention away from the causal effect of the exposure and instead focus on how much disparity due to a risk factor would remain if a potential intervention would target a downstream mechanism [2, 3]. This health interventional disparity approach—the idea of estimating changes in health outcomes due to an intervention on a manipulable and specific mediator, has been applied to socially determined characteristics in epidemiology [4, 5]. In recent years, genetic data of research participants has become increasingly available in large epidemiological studies. In this paper, our aim is to apply the health disparity interventional approach to differences in health outcomes attributed to differences in genetic risk. We hope that this framework might offer new opportunities for researchers in genetic epidemiology and causal inference, grounding research questions within public health, by asking researchers to hypothesise potential interventions to mitigate disparity associated with genetic risk.

### Genetically informed designs to study causation

Researchers have exploited genetically informed research designs to study causation, for example analysing datasets with related individuals such as twins [6]. These research designs, which include prior knowledge of the participants’ genetic relatedness allow researchers to capture some of the genetic and environmental unmeasured confounding, which can strengthen causal inferences. Using twin and sibling designs researchers have studied the causal relationship between exposures and health outcomes such as socio-economic position and depression [7], bullying victimisation and self-harm [8], as well as smoking and lung cancer [9]. Building on family-based studies, technological advances have allowed the mass collection of DNA from participants, and genotypes are now commonly available in large-scale population cohorts. This enables researchers to construct genetic liability indicators for common health outcomes and has led to the integration of genetic data into causal inference within epidemiological research [10]. Well-known examples are the numerous applications of Mendelian Randomisation, which uses genetic variants as instruments to estimate causal effects [11, 12]. Further, genetic data have been integrated into longitudinal studies of families and their children, allowing a more robust study of intergenerational genetic and environmental effects [13, 14].

### Genes, polygenic scores, and counterfactuals

Fundamental to this research, is the assumption that genomic variants *cause* variations in phenotypes. This central dogma of biology describes a one-way stream of information, whereby variations in DNA result in differences in RNA, which in turn are responsible for the synthesis of proteins [15, 16], essential to biological functioning and development. Historically, this central dogma, developed at the same time as the emergence of computer science. Their synergies popularised commonly used terms such as the “genetic code” and the DNA as “blueprint” of life, “transcribing” and “translating information” [17, 18]. In the light of the current scientific consensus, different meanings of genes, or genetic effects, need to be considered when aiming to conceptualise genetic information as exposures in causal inference. As described in detail by Lynch (2021), genetic research, can be crudely categorised into questions regarding monogenic or polygenic traits. The former considers that changes in a singular gene cause changes in the product of the coded protein. In contrast, the latter refers to the fact that most common phenotypes are influenced by many genetic markers (polygenicity) which in turn influence many different outcomes (pleiotropy) [19]. These two theoretical settings need to be carefully considered when using genetics to study causal mechanisms over the life course. Within the stricter definitions of causality, an exposure of interest needs to be intervenable, and result in a specific causal effect [1]. In the context of mono-genic traits, and the advent of gene editing technology, such as a CRISPR, interventions on mono-genic traits could be deemed as acceptable [20]. However, for polygenic traits this might be harder to argue, as these often relate to common health outcomes, which are influenced by 1000s of genomic variants with pleiotropic effects [21]. This pleiotropy obscures the causal chain between polygenic score (PGS), which summarises the additive effects of multiple variants into one singular score, and outcome, leading to a broad rather than specific effect. Lastly, PGS might not be considered as feasible potential intervention targets. Embryo selection based on polygenic scores for common illnesses is currently not plausible and poses difficult ethical questions, as outcomes of such interventions will have unknown, potentially adverse and wide-ranging effects [22, 23]. Regardless, PGS have been described as promising tools in precision medicine and are commonly used in epidemiological research [24], but at the same time have been criticised for the their lack of theoretical and empirical evaluation [25, 26].

### Health disparity measure approach

**Fig. 1:**
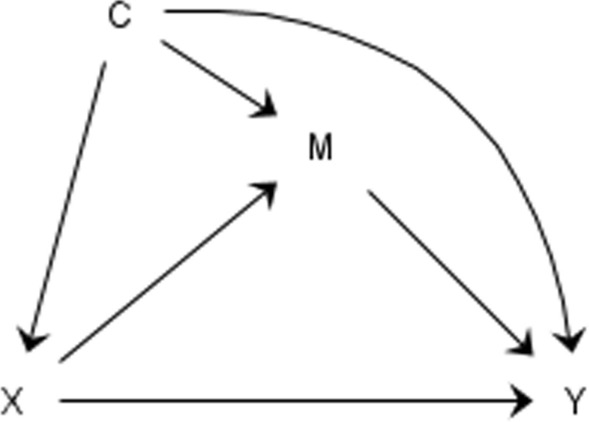
Mediation diagram with a direct effect from exposure (X) to outcome (Y), and indirect effects via an intermediate mediator (M), adjusted for confounder (C)

In summary monogenic traits may be conceptualised as causes within the stricter definitions of causal inference, however, this might not apply to polygenic traits. Therefore, in this paper, our goal is to borrow the idea of health disparity interventional affects and apply this framework to PGS, building on the previous work of our group [27]. Health disparity measures can be best understood in the context of mediation analysis where the focus is to identify whether some of the total effect between exposure (X) and outcome (Y), works via an intermediate mediator (M) (see Fig. 1). The total causal effect is defined by imagining the potential outcome of a hypothetical intervention on the exposure (X), comparing the outcome if the participant was exposed (e.g. X = 1) versus the potential outcome if the participant was not exposed (X = 0). This principle is then extended to causal inference mediation models, where the hypothetical intervention targets both the exposure and the mediator [28, 29]. When hypothetical interventions on the exposure are not justifiable, interventional disparity measures can be useful to express how much of the association between exposure and outcome would remain if we intervened on the distribution of the mediator. Here, we describe how this approach can be applied to PGS and then follow with examples using data from the Avon Longitudinal Study of Parents and Children and the Millennium Cohort Study, both from the United Kingdom.

## Methods

The health disparity measure approach focuses on two estimands: the Interventional Disparity Measure—Direct Effect (IDM-DE) and the Adjusted Total Association (Adj-TA). As outlined by Micali et al. (2018), the Interventional Disparity Measure—Direct Effect (IDM-DE) is the disparity in the outcome (Y) associated with the exposure that remains if we were to intervene on the intermediate mediator (M), by shifting the distribution of M to the distribution that M would have had under no exposure (X = 0) [29].

In the situation of a binary exposure, and continuous mediator and outcome, we can specify ${M}_{C}^{0}$ as a random draw from the distribution of M conditional on the confounder C when X is set to take the reference value 0. Let Y(m) be the potential outcome when the mediator M is set to take the value m, in this case taking the randomly drawn value ${M}_{C}^{0}$. The IDM-DE is defined as:

$$\text{IDM-DE}=\sum_{c}\begin{array}{c}[E\{Y({M}_{C}^{0})|X=1 , C=c \}-\\ E \{Y({M}_{C}^{0})|X=0, C=c\}] Pr(C=c)\end{array}$$

where C is here assumed to be categorical. For general definitions see Daniel and De Stavola (2019) [30]. In addition to the IDM-DE, we also aim to estimate the association between exposure and outcome, without intervening on the mediator M. This Adj-TA is defined as:

$$\text{Adj-TA}=\sum_{c}\begin{array}{c}[E\{Y|X=1, C=c \}-\\ E\{Y|X=0, C=c\}] Pr\left(\mathrm{C}=\mathrm{c}\right),\end{array}$$

The difference between Adj-TA and IDM-DE gives some indication of the potential change in disparity due to the hypothetical intervention.

These general definitions apply to situations with a binary exposure. However, genetic liability is commonly expressed as a continuous PGS. In this scenario, one option would be splitting the distribution of the exposure into multiple equal sized groups, representing the participants ranging from low to high genetic liability, for example, sample size permitting, quintiles (1 = lowest risk, 2 = lower risk, 3 = average risk, 4 = high risk, 5 = highest risk; indexed by j). In this case, the definitions of the IDM-DE and Adj-TA need to be adapted, depending on the choice of reference category. Let ${M}_{C}^{1}$ be a random draw from the distribution of M conditional on the confounder C when X is set to take the reference value 1. The disparity measures of interest are then defined as, for j = 2,3,4,5,

$${\text{IDM-DE}}_{j}=\sum_{c}\begin{array}{c}[E\{Y({M}_{C}^{1})|X=j , C=c \}-\\ E \{Y({M}_{C}^{1})|X=1, C=c\}] Pr(C=c),\end{array}$$

The same applies for the Adj-TA, at each level of the exposure in reference to the lowest liability reference (j = 1). This is defined as, for j = 2,3,4,5

$${\text{Adj-TA}}_{j}=\sum_{c}\begin{array}{c}[E\{Y|X=j, C=c \}-\\ E\{Y|X=1, C=c\}] Pr\left(\mathrm{C}=\mathrm{c}\right),\end{array}$$

Estimation of the interventional disparity measures call upon the three assumptions of no interference, consistency, and no unmeasured confounding of mediator-outcome associations [25].

## Motivating example

We aim to study the extent to which a potential intervention on physical activity could mitigate genetic liability for obesity in childhood. Childhood obesity remains one of the main health concerns globally [31]. Children with overweight or obesity have been found to show higher risk of overweight and obesity in adulthood, which is associated with other health outcomes, such as cancer, depression, and asthma [32–34]. Further, individuals with overweight and obesity face bullying and stigmatisation from their peers and health professionals, contributing to the health burden [35, 36]. Individual differences in body size have been studied extensively, and twin [37] and genome-wide association studies have provided evidence for a substantial genomic contribution, indicating that hundreds if not thousands of genetic variants are associated with BMI [38]. In addition, rapid changes in the food environment (larger portion sizes, availability of high fat foods) as well as life-style changes (sedentary work and leisure) have been identified as risk factors [39]. Here we imagine an intervention on a downstream behavioural factor, physical activity, which might mitigate some of the genetic liability. Physical activity has been targeted in randomised intervention trials for childhood obesity [40, 41], and genomic studies have suggested that a higher genetic liability for obesity is associated with lower physical activity [42].

**Fig. 2:**
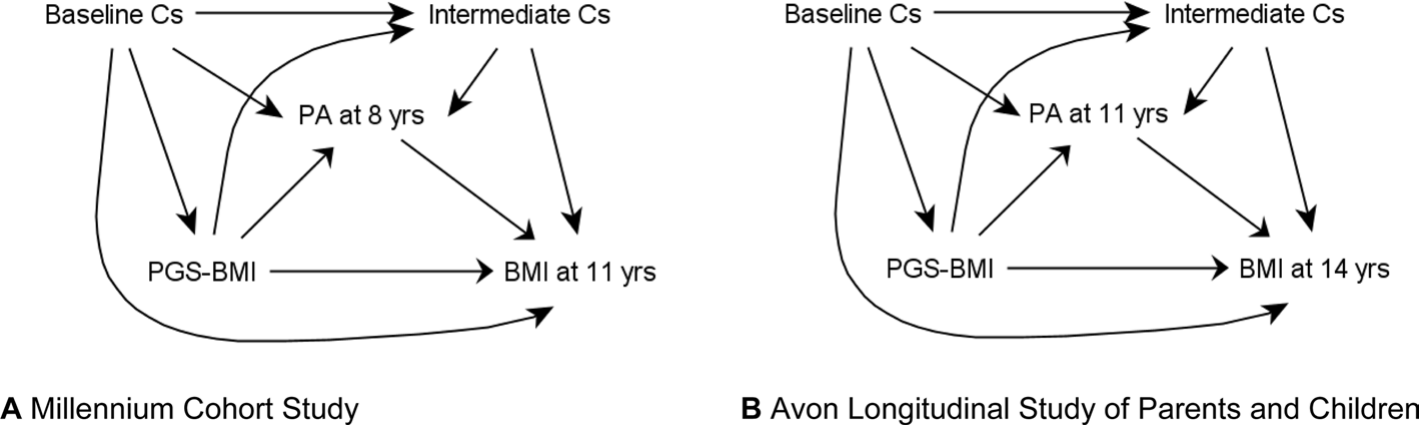
(**A** and **B**) Diagram of mediation model using data, baseline covariates include child sex, maternal education at birth and maternal pre-pregnancy BMI. Additionally, potential intermediate confounders are also depicted and could be childhood (respectively pre-age 8 and pre-age 11) behavioural and metabolic factors, e.g. diet, or living in an urban environment. *PGS-BMI* polygenic score BMI, *Cs* confounders, *PA* physical activity, *BMI* body mass index, *yrs* years

In the following, we will estimate the IDM-DE and the Adj-TA to understand the extent to which an intervention on physical activity in childhood can mitigate the association between genetic liability measured by a PGS and later BMI. Data are from the Avon Longitudinal Study of Parents and Children (ALSPAC) [43] and the Millennium Cohort Study (MCS) [44]. Full details of the samples and measurements can be found in *Supplement Text* and *Supplement Table 1.* In short, in both cohorts PGS for BMI were calculated using the conditional shrinkage method, developed by Ge et al. [45] from summary statistics of the Genetic Investigation of Anthropometric Traits (GIANT) consortium [38], using the automated analyses pipeline GenoPredPipe [46]. The PGS was then categorised using cohort-specific quintiles. Physical activity was measured using accelerometers when the children were 8 (MCS) or 11 years (ALSPAC), indicating the average minutes of moderate to vigorous physical activity (MVPA) over the course of one week. BMI measures were obtained during research clinic visits at age 11 years (MCS) and 14 years (ALSPAC). Included confounders were maternal education, maternal BMI prior to pregnancy, and child sex using parental report. The hypothesised associations are outlined in Fig. 2a (MCS) and Fig. 2b (ALSPAC). The analyses sample sizes were 2575 and 3347 for MCS and ALSPAC respectively and included complete cases only, followed by sensitivity analyses using imputation.

## Estimation

Analyses, consisting of a series of regressions for the mediators and outcomes, were conducted in Stata version 16, with estimation of the IDM-DE and the Adj-TA carried out by plug-in parametric estimation and Monte Carlo simulation on a 1000-fold expanded dataset, with 1000 bootstrap samples to derive confidence intervals. Regression models included non-linear terms and interactions between confounders and mediators to allow for general parametric specifications and thus avoid unnecessary restrictive assumptions (e.g. with respect to linearity of associations).

## Sensitivity analyses

In both studies, data on exposure, confounders, and mediators were affected by missingness. For this reason, the Monte Carlo estimation procedure described above was repeated after implementation of a single stochastic imputation of the missing values, using chained equations (with 10 burn-in iterations) assuming missingness was at random (given the observed data). The imputation models included all variables that contributed to the analytical models allowing for non-linearities and interactions. Standard errors were again estimated via bootstrap (with the imputation step redone on each bootstrap sample), avoiding the need for multiple imputations.

To examine the impact of unmeasured mediator-outcome confounders, we used an approach first suggested by Imai et al. [47] and then expanded in De Stavola et al. [48] which consisted in estimating the minimal size of the correlation induced by a confounder that, if controlled for, would remove the impact of the mediator. This correlation is reported for each study, with bootstrapped 95% confidence intervals.

Analyses were pre-registered and the code for all the analyses is available, see https://osf.io/9hbmu/.

**Fig. 3:**
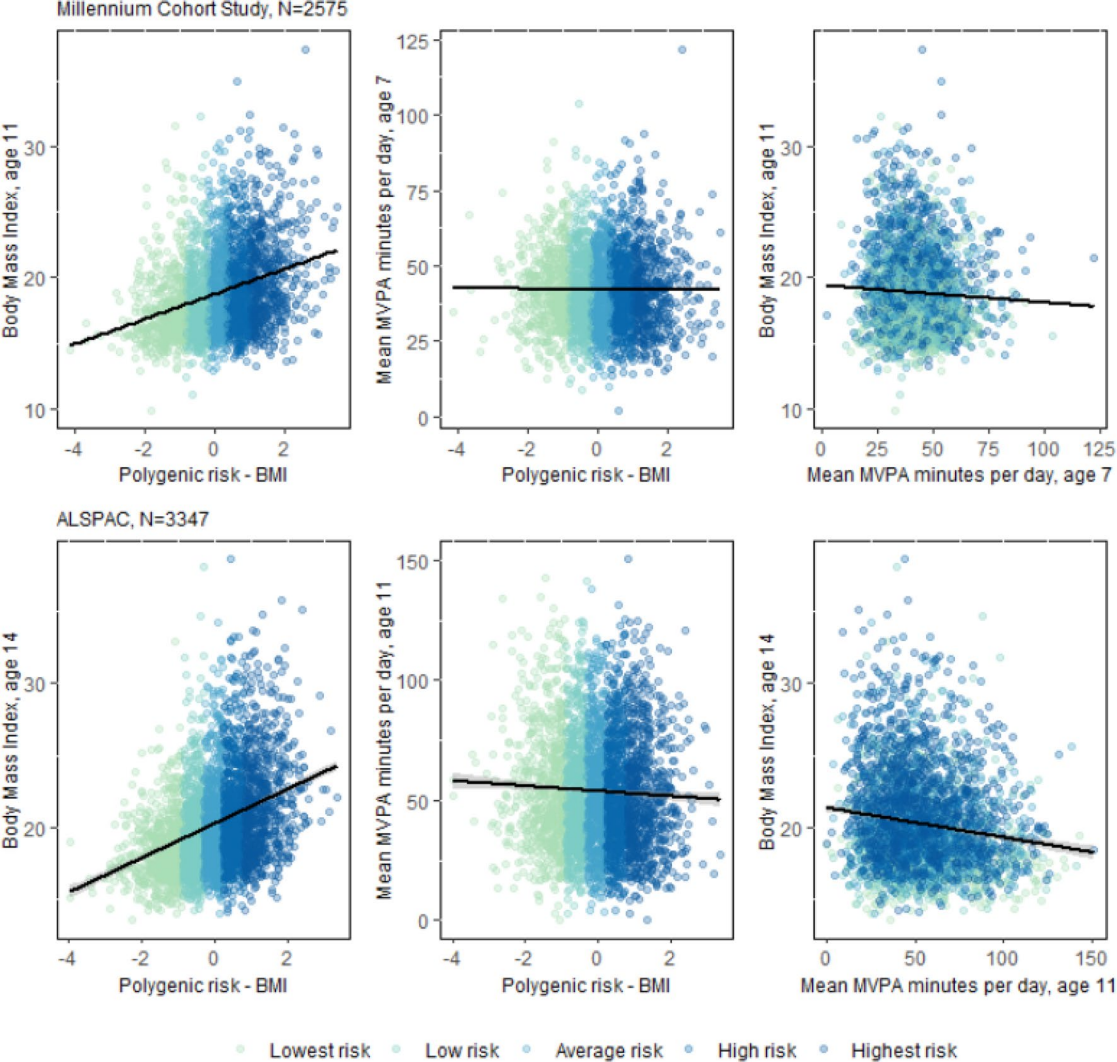
Correlation plots for the exposure, mediator and outcome variables in the Millennium Cohort Study and Avon Longitudinal Study of Parents and Children. Colours indicate the BMI-PGS quintiles. *MVPA *moderate to vigorous physical activity. Black lines show the fitted regression lines

Figure 3 shows the scatter plot of childhood BMI (the outcome) and physical activity (the mediator) against PGS-BMI (the exposure), with points colour-coded to reflect PGS quintiles. The lines show the predicted regression lines. These figures give a visualisation of the unadjusted associations between exposure, mediator, and outcome. The PGS-BMI is positively associated with BMI, and negatively associated with physical activity. BMI and physical activity are negatively associated. For more detailed information, means of MVPA and BMI, in each PGS-BMI quintile and their correlations are listed in Supplement Tables 2 and 3.

**Fig. 4:**
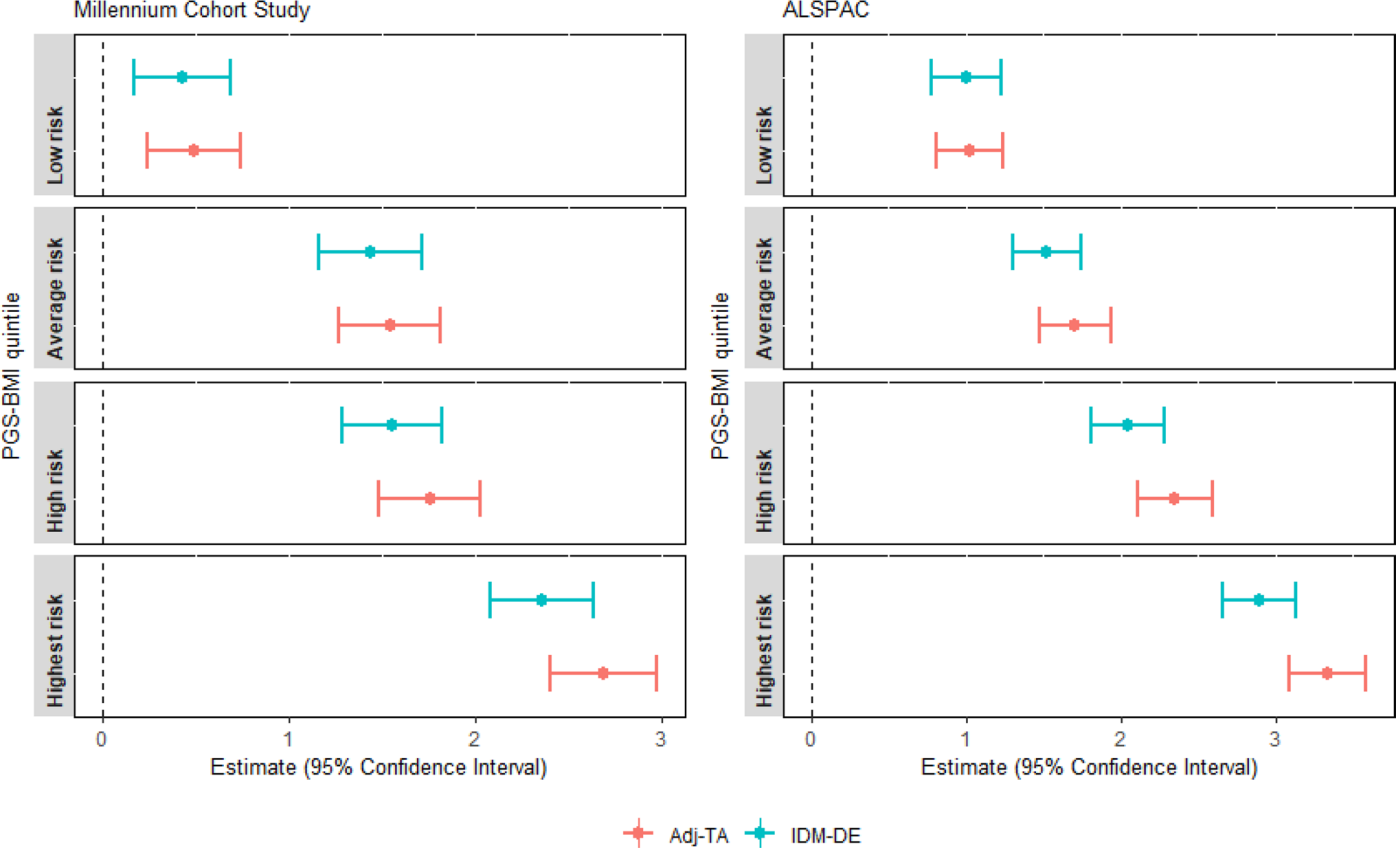
Adjusted total association (Adj-TA) and Interventional Disparity Measure—Direct Effect (IDM-DE), given a hypothetical intervention shifting the distribution of moderate physical activity to the distribution under lowest genetic risk (reference category lowest risk, not depicted), in the Millennium Cohort Study (N = 2575) and the Avon Longitudinal Study of Parents and Children (ALSPAC, N = 3347)

The hypothetical physical activity intervention envisaged here, shifts the distribution of physical activity to that experienced by those in the lowest genetic quintile (conditional on confounders; coloured in light green in Fig. 3). The resulting IDM-DEs and Adj-TAs are shown in Fig. 4. Estimates and 95% confidence intervals are listed in Supplementary Table 4a and b. Overall, the hypothetical interventions to shift the four top strata defined by categorical PGS-BMI to mirror the distribution in the lowest PGS-BMI category have a small impact on the total association between PGS and later BMI. This is indicated by the small or no differences between the IDM-DE and the Adj-TAs (see Fig. 4). The biggest differences are found for the highest PGS quintile (dark blue colour in Fig. 3), whereby the change in physical activity to what it would have been under lowest PGS quintile, was estimated to remove 0.33 kg/m^2^ (95%CI 0.21, 0.44) in BMI at 11 years in MCS (Adj-Ta = 2.69, 95%CI 2.40, 2.98; IDM-DE = 2.36, 95%CI 2.08, 2.64). In ALSPAC, the results followed a similar pattern, whereby the potential intervention was associated with a difference of 0.44 kg/m^2^ (95%CI 0.32, 0.56) in BMI at 14 years (Adj-Ta = 3.34, 95%CI 3.09, 3.59; IDM-DE = 2.9, 95%CI 2.66, 3.14). For the other quintiles of PGS, the impact of their respective interventions was smaller in both MCS and ALSPAC. Note that the interventions applied to each PGS-BMI quintile is of different magnitude because the shift in physical activity decreases from the highest to the first category (see Supplementary Table 3).

## Sensitivity analyses

Estimates of the differences between the Adj-TA and the IDM-DE obtained from the imputed data (respectively based on N = 6172 in MCS and N = 6035 in ALSPAC) became slightly larger than those obtained from the complete records only (MCS, N = 2757 and ALSPAC, N = 3347). For comparison, the estimated differences in MCS were 0.06, 0.10, 0.21 and 0.33 when using the complete records and 0.08, 0.10, 0.21 and 0.34 when using the imputed data. In ALSPAC the estimated differences from complete records were 0.02, 0.18, 0.30 and 0.44 from the complete records and 0.11, 0.28, 0.35 and 0.65 from the imputed data.

The separate estimates of the Adj-TA and the IDM-DE were also slightly larger when using the imputed data. This might reflect the selection in participation by socio-economic position which is known to affect ALSPAC in particular [49]. A full list of the estimates from the imputed data can be found in Supplementary Table 4c and d.

Examining the potential consequences of unmeasured confounding between mediator and outcome in both studies, we found that the impact of intervening on the mediator would be null if there were an additional confounder to those included in the analyses that induced a correlation between physical activity and BMI (above that induced by the measured confounders) of − 0.11 (95%CI − 0.13, − 0.07) in ALSPAC and − 0.03 (95%CI − 0.07, 0.01) in MCS. These estimates reflect the weak and nearly null results found in the two studies.

## Discussion

In this paper, we aim to demonstrate how polygenic scores within the interventional disparity approach could be used in the context of causal inference analysis. This approach is proposed as a novel additional tool for genetic epidemiological researchers with an interest in public health. Building on some previous work [27], these results suggest that a hypothetical intervention increasing physical activity has the potential to buffer a small proportion of disparity in BMI associated with the BMI PGS. This small impact needs to be considered in the context of randomised control trials (RCT) of interventions for childhood obesity. A meta-analysis of 14 RCTs indicated that physical activity interventions result on average in a BMI reduction of 0.10 kg/m^2^, and that the most effective interventions combined physical activity and dietary components [41]. Further, it is important to emphasise that our hypothetical intervention does not estimate the effect of changing physical activity on later BMI, but the extent to which intervening on physical activity can mitigate the association between genetic liability and BMI. Our analyses do not aim to find the intervention that is associated with biggest decrease in BMI or of increasing physical activity to its most beneficial level. Instead, we aim to investigate the extent to which potential interventions that improve the distribution of physical activity of individuals with high genetic liability, would remove part of their liability. We hope to have demonstrated how the health disparity measure interventional approach can be applied to estimate the potential impact of hypothetical interventions to reduce the disparity associated with genetic risk. Results from analyses of our two datasets produced only small differences between the adjusted total effect versus the interventional direct effect, but it should be noted that the shift of distribution considered in our calculations is driven by the strength of association between the PGS and the mediator. Other settings involving different PGS, mediators, and outcomes, might lead to greater shifts and hence greater disparity reductions. One additional benefit of the health disparity approach is that it asks researchers to specify a clear potential intervention target, grounding research in real life and pushing us to think through the implications and feasibility of the hypothetical intervention.

### Health inequalities and genetics

Health inequalities are commonly understood as differences in health outcomes due to determinants that are outside of the individual’s control, which could be remedied by policy intervention. For example, there have been established observations that socio-economic position at birth can result in longstanding negative health outcomes [50]. Childhood obesity rates are highest in families with lowest income and education levels [51], and policy interventions have aimed to close this gap, targeting individual behaviours (e.g. healthy foods in schools programme) as well as structural (e.g. taxation on high energy dense foods) components with limited success [52]. More recently there has been a call to broaden health inequality exposures and to consider the question of whether genetic propensity for a health outcome can be considered as a cause for health inequalities. This might be seen as intuitive, as genetic factors are associated with later health outcomes and are also outside of an individual’s control [53]. However, a debate is still ongoing if these genetic differences in the population should be included in the study of social determinants of health [54]. Targeting individuals based on their genetic liabilities has been argued to be a direct continuation of the horrific eugenic practices of the last century [55], and there is a need for an ethical and theoretical framework on how to regulate the already existing embryo screening technology which uses PGS [23].

### Assumptions and considerations

Most causal inference methods lean on the three major assumptions of no interference, consistency, and no unmeasured confounding. Interference would be present if the intervention target for one participant would impact the outcome in another participant. For example, behaviours of one participant might influence another, if the two participants are in the same class in school, or maybe members of the same extended family. In our analysis, this situation might be considered as highly unlikely, as the participants of cohort studies are commonly recruited from a large region. Further, it might be recommended to only include one participant per family, excluding siblings and cousins, which is what we have done here. It should be noted that checks for genetic relatedness based on observed genomic data are common practice in the quality control when analysing genetic data. The consistency assumption implies that the distributional intervention is “non-invasive” meaning that the outcomes for the participants would not have differed had they been observed or intervened to have that mediator distribution [56, 57].

One additional limitation is the weak association between the exposure and the mediator, found in both studies. The size of these associations clearly binds the scale of change due to the hypothetical interventions and demonstrates the challenges of studying intervenable pathways from genomic liability to a later outcome. Further applications of this framework should select mediators with stronger associations with the polygenic score of interest.

Lastly, the no unmeasured confounding assumption of the mediator-outcome association cannot be formally tested. However, we included baseline covariates, and they might capture at least some of the confounding. As illustrated in Fig. 2a and b, there is potential for unmeasured intermediate confounding of the mediator to outcome associations affecting our analyses, for example via dietary factors or environmental factors such as urban environment, for which we do not have reliable information. This may have led to negative confounding and hence overestimation of the interventional effects, in the presence for example of a negative association of urban environment with physical activity and positive association with BMI. Our sensitivity analyses showed that in ALSPAC such correlation would need to be at least − 0.1. Including participants with complete data on exposure, mediator, outcome, and all covariates resulted in reduced sample sizes, as well as the potential introduction of selection bias, as participants with complete records are those who contributed data to all relevant collection waves and these individuals may differ from the rest of the original cohort members. However, imputation, under the missing at random (MAR) assumption, did not lead to substantially different results.

Additionally, researchers aiming to apply this framework, need to be aware of the pitfalls that underlie the construction and interpretation of PGS. PGS aggregate effect sizes associated with single nucleotide polymorphism, but do not (yet) include other type of genomic variations, such as rare variants, deletions, and copy number variations. Hence, PGS only capture a proportion of the variance in the outcome. This incomplete measure of genetic liability has been shown to have consequences for mediation analyses, specifically leading to an exaggeration of the indirect effect, from genetic liability to outcome, via a mediator [58]. Further limitations in this area that remain are that, even though sample sizes have grown rapidly, the majority of available summary statistics are based on participants of white European descent which cannot be readily transported to global and diverse population cohorts [59].

As highlighted above, effective public health interventions for multifactorial diseases such as obeisty are most likely to target a complex combination of social and behavioural changes, e.g. diet, physical activity and parenting behaviours. However, our aim is to demonstrate how to quantify how much of the genetic effect of the BMI PGS would remain if its effect on PA were "equalised". Here we consider a single possible area of intervention, physical activity, to examine interventional effects, but methods for multiple mediators are available [28] and future work should aim to explore these in the context of genetically informed studies.

### Conclusions

We have provided an example of how the health disparity framework might be implemented in longitudinal cohorts with genetic, behavioural, and anthropometric data. This approach lends itself to many non-communicable health outcomes that have some genetic aetiology but whose interventions often require changing behavioural or environmental targets. We see this approach in addition to methods applied to gene-environment interplay but grounded by the formal restrictions of specifying a plausible intervention target, linking research directly to questions of public health relevance.

## Supplementary Information

Below is the link to the electronic supplementary material.

Supplementary file1 (DOCX 62 kb)
