# Supplementary material for: Could interventions on physical activity mitigate genomic liability for obesity? Applying the health disparity framework in genetically informed studies
Source: Eur J Epidemiol. 2023 Mar 11;38(4):403–12. doi: 10.1007/s10654-023-00980-y (PMC10082115; doi:10.1007/s10654-023-00980-y)
Supplement: Supplementary file 1 — Supplementary file1 (DOCX 62 kb) [file 10654_2023_980_MOESM1_ESM.docx]

SUPPLEMENTAL INFORMATION

**Sample**

*The Avon Longitudinal Study of Parents and Children*

The Avon Longitudinal Study of Parents and Children (ALSPAC) recruited pregnant women in the southwest of England (24, 25). All pregnant women that were expected to have a child in the period of 1 April 1991 until 31 December 1992 were contacted to participate in the original cohort. At the beginning, 14,451 pregnant women took part, and 13,988 children were alive at the end of year one. To guarantee independence of individuals, one sibling per set of multiple births (n = 203 sets) is randomly included in our sample. Please note that the study website contains details of all the data that are available through a fully searchable data dictionary and variable search tool and reference the following webpage: http://www.bristol.ac.uk/alspac/researchers/our-data/.

Ethical approval for the ALSPAC participants was obtained from the ALSPAC Ethics and Law Committee and the Local Research Ethics Committees: www.bristol.ac.uk/alspac/researchers/research-ethics/. Consent for biological samples was collected in accordance with the Human Tissue Act (2004). The analyses sample only consisted of participants with complete data on exposure, mediator, outcome and covariates.

*Millennium Cohort Study*

The UK Millennium Cohort Study (MCS) is a longitudinal cohort study of over 19,000 children both in the UK between 2000 and 2002 [44]. These analyses include measures from baseline, wave 4 (7 years) and wave 5 (11 years).

The Millennium Cohort Study was approved by the South West, London and Yorkshire Multi-Centre Research Ethics Committees (MREC/01/6/19, MREC/03/2/022, 05/MRE02/46, 07/MRE03/32, 11/YH/0203, 13/LO/1786for sweeps one, two, three, four, five and six respectively). The waves reported were conducted under relevant ethical committee approval from the National Health Service (NHS) Research Ethics Committee system which are appointed by the Strategic Health Authorities in England; parental written, informed consent was obtained of all children.

**Measures**

*BMI*

Millennium Cohort Study

Body mass index at 11 years was calculated from measures of height and weight. Children were weighed without shoes or outdoor clothing by trained interviewers using Tanita HD305 scales (Tanita UK Ltd, Middlesex, UK). Weights were recorded in kilograms to one decimal place. Heights were measured with the Leicester Height Measure Stadiometer (Seca Ltd, Birmingham, UK) and recorded to the nearest millimetre.

The Avon Longitudinal Study of Parents and Children

Height and weight were measured during clinic visits when the children were 14 years old. Weight was measured with a Tanita Body Fat Analyzer (Tanita TBF UK Ltd) to the nearest 50g. Height was measured to the nearest millimetre with the use of a Harpenden Stadiometer (Holtain Ltd). BMI was calculated by dividing weight (in kg) by height (in m) squared.

*Physical activity*

Millennium Cohort Study

Physical activity was measured with accelerometers at 8 years using Actigraph GT1M accelerometer (Actigraph, Pensacola) worn for 7 days. A valid day was defined as providing data for at least 10 hours per day (excluding sequences of 10 or more minutes with consecutive zero counts) and data was deemed valid if participants provided at least 2 valid days of recording. Total physical activity is the total volume activity including activities at all intensities, measured as the average counts per minute (cpm) over the period of valid recording. Daily average of moderate-to-vigorous physical activity (MVPA) was defined as >2241 counts per min (cpm), following standard procedures.

The Avon Longitudinal Study of Parents and Children

Physical activity was measured with accelerometers at 11 years. Participants were asked to wear an Actigraph AM7164 2.2 accelerometer (Actigraph LLC, Fort Walton Beach, FL, USA) around their waist, at the right hip, for 7 days. A valid day was defined as providing data for at least 10 hours per day (excluding sequences of 10 or more minutes with consecutive zero counts) and children were only included in the analyses if they provided at least 3 valid days of recording. Following variables were derived from the raw measures. Total physical activity is the total volume activity including activities at all intensities, measured as the average counts per minute (cpm) over the period of valid recording. MVPA minutes per day were calculated using a who calculated a lower cutpoint of >=2296 cpm following Evenson et.al (2008) [60].

*Polygenic risk score – BMI*

In both samples polygenic score BMI was calculated using the Bayesian shrinkage approach by Ge et al, which uses a high-dimensional Bayesian regression framework, that includes a continuous shrinkage prior on the effect sizes of the included Single Nucleotide Polymorphisms (SNPs). This method has the advantage of allowing researchers to add all potential SNPs into the PGS, without clumping or choosing a p-value threshold to specify inclusion [45]. EUR 1000 genomes panel was used as LD reference, and the automatic option was used to specify the shrinkage parameter, which estimates the optimal shrinkage parameter from the summary statistics. This method has been found to be superior in comparison to some of the other polygenic scoring methods, as it is able to explain the greatest amount of variance [46]. The PGS was calculated using GenoPredPipe – an analyses pipeline which automates data cleaning, curation, and calculation of polygenic scores within a standardised reference framework. GenoPredPipe is a snakemake pipeline implementing reference-standardised polygenic scoring [46]. Full documentation of the pipeline can be found online (<https://opain.github.io/GenoPred/>).

The Avon Longitudinal Study of Parents and Children

Genotype data were available for 9,915 children out of the total of 15,247 ALSPAC participants. Participants were genotyped on the genome-wide Illumina HumanHap550 quad chip. Pre-processing of the extracted data include stringent quality control procedures. Individuals with disproportionate levels of individual missingness (i.e., >3%), insufficient sample replication (identity by descent < 0.8), biological sex mismatch, and non-European ancestry (as defined by multi-dimensional scaling using the HapMap Phase II, release 22, reference populations) were excluded. SNPs with a minor allele frequency (MAF) of < 1%, excessive missingness (i.e., call rate < 95%), or a departure from the Hardy–Weinberg equilibrium (P value < 5 x 10-7) were removed. Imputation was conducted with Impute3 using the HRC 1.0 as the reference panel and phasing was carried out using ShapeIT (v2.r644). Finally, post-imputation quality control checks were performed; any SNPs with MAF less than 1%, Impute3 information quality metric of < 0.8, and not confirming to Hardy-Weinberg equilibrium (P < 5 × 10-7) were removed. After data cleaning, a total of 8,654 individuals remained eligible for analyses.

Millennium Cohort Study

Saliva samples for DNA genotyping were collected when participants were 14 years old, using Oragene DNA Self-Collection Kits OG-500. 11,806 participants were eligible, and 9259 (78%) supplied valid consent and DNA was extracted successfully. Genotyping of samples was conducted using Infinium global screening arrays-24 v1.0 from Illumina, with 24 samples on each chip. Procedures were done following manufacturer's instructions. Genotype calling was performed using Genome Studio v2.0.4. Data for 618,540 genetic variances was then quality controlled using QCtools_v2.0.1, resulting in 602,181 SNPs passed for 8173 participants. Only participants coded as

*Covariates*

In both samples, included covariates were child sex at birth, self-reported maternal BMI prior to pregnancy. High maternal education at birth of child was defined by mothers having completed education up to A-Levels, the requirement for applying to university in the UK.

**Supplement Tables**

Supplement Table 1: Descriptive characteristics of the analysis samples

|  | | **Avon Longitudinal Study of Parents and Children** | | **Millennium Cohort Study** | |
| --- | --- | --- | --- | --- | --- |
| **Characteristics** | | **15,645 total sample,**  **15,442 singletons,**  **14,683 Alive at 1 year** | **Analyses sample, complete records n=3,347** | **9,291 total participants,**  **8,030 first child per family** | **Analyses sample,**  **complete records, n= 2,575** |
|  |  | **Mean (SD) or n (%)** | | | |
| **Sex** | Boys  Girls | 7,489 (51%)  7,194 (49%) | 1,564 (46%)  1,815 (54%) | 3,835 (50%)  3,822 (50%)  n=7,657 | 1,258 (49%)  1,317 (51%) |
| **Maternal education** | < A-levels  > A-levels | 7,915 (65%)  4,328 (35%),  n=12,243 | 1,823 (54%)  1,556 (46%) | 3,399 (52%)  3,822 (48%)  n=6,517 | 1,156 (45%)  1,419 (55%) |
| **Maternal BMI before pregnancy** |  | 22.9 (3.9),  n=11,388 | 22.9 (3.7) | 23.9 (4.5),  n=7,082 | 23.9 (4.2) |
| **Child ethnicity** |  | White=11,838 (97%)  Black Caribbean= <1%  Black African= <1%  Other black= <1%  Indian= <1%  Pakistani= <1%  Bangladeshi= <1%  Chinese= <1%  Other= <1% | White participants = 3,347 (100%) | White= 6,569 (84%)  Mixed= 196 (3%)  Indian=193 (3%)  Pakistani and Bangladeshi = 517 (7%)  Black and black British= 169 (2%)  Other ethnic group (inc Chinese)=104 (1%) | White participants  = 2,575 (100%) |
|  | **Exposure** | | | | |
|  |  | **PGS-BMI** | | **PGS-BMI** | |
|  |  | 0.00 (1.0),  n=8.805 | -0.04 (1.00) | 0.2 (1.09),  n=6,831 | 0.1 (1.07) |
|  | **Mediator** | | | | |
|  |  | **Mean MVPA per day, 11 years** | | **Mean MVPA per day, 8 years** | |
|  | | 56.7 (30),  n=5,878 | 53.9 (23.9) | 42.4 (13.1),  n=4,155 | 42.3 (12.9) |
|  | **Outcome** | | | | |
|  |  | **BMI, 14 years (kg/m^2^)** | | **BMI, 11 years (kg/m^2^)** | |
|  |  | 20.4 (3.5),  n=6,035 | 20.3 (3.4) | 19.2 (3.6),  n=7,498 | 18.9 (3.3) |

MVPA = Moderate to vigorous physical activity

Supplement Table 2 Correlation table (95% Confidence intervals) for exposure, mediator, and outcome in the two cohorts

|  | Millennium Cohort Study  (n= 2,575) | | |
| --- | --- | --- | --- |
|  | PGS-BMI | MVPA 8 years | BMI 11 years |
| PGS-BMI | 1 |  |  |
| MVPA 8 years | -0.002  (-0.04, 0.04) | 1 |  |
| BMI 11 years | 0.31  (0.28, 0.35) | -0.05  (-0.09, -0.01) | 1 |
|  | Avon Longitudinal Study of Parents and Children  (n=3,347) | | |
| PGS-BMI | 1 |  |  |
| MVPA 11 years | -0.04  (-0.08, -0.01) | 1 |  |
| BMI 14 years | 0.36  (0.33, 0.39) | -0.15  (-0.18, -0.11) | 1 |

Abbreviations: MVPA = Moderate to vigorous physical activity, PGS-BMI = Polygenic score – Body Mass Index, BMI = Body Mass Index

Supplement Table 3. Mean and standard deviations for moderate-to-vigorous exercise per day and BMI across the genetic liability quintiles

|  | **Millennium Cohort Study**  n=2,575 | | **Avon Longitudinal Study of Parents and Children**  n=3,347 | |
| --- | --- | --- | --- | --- |
| **PGS-BMI quintile** | **MVPA mean minutes per day at 8 years** | **BMI at 11 years** | **MVPA mean minutes per day at 11 years** | **BMI at 14 years** |
| **1** | 42.4 (12.6) | 17.5 (2.7) | 55.7 (25.2) | 18.5 (2.3) |
| **2** | 42.4 (12.5) | 18.0 (2.7) | 53.9 (23.2) | 19.7 (2.9) |
| **3** | 42.2 (12.7) | 19.1 (3.2) | 53.3 (23.2) | 20.4 (3.2) |
| **4** | 42.0 (12.8) | 19.3 (3.3) | 53.6 (23.5) | 20.9 (3.5) |
| **5** | 42.3 (13.8) | 20.3 (3.5) | 52.3 (23.8) | 21.9 (3.5) |

Abbreviations: MVPA = Moderate to vigorous physical activity, PGS-BMI = Polygenic score – Body Mass Index, BMI = Body Mass Index

Supplement Table 4a. Interventional Disparity Measure – Direct Effect (IDM-DE) and adjusted total association (Adj-TA) of categorical PGS-BMI versus the reference category of lowest genetic risk using Millennium Cohort Study data: estimates and 95% Confidence intervals, n= 2,575

| **PGS-BMI** | **Estimate** | | | **95% CIs** | | **Difference** | **95% CIs** | | |
| --- | --- | --- | --- | --- | --- | --- | --- | --- | --- |
| **Lowest risk** | **Reference** | | | | | | | | |
| **Low risk** | **IDM-DE_2_**  **Adj-TA_2_** | \| 0.43 \| \| --- \| \| 0.49 \| | 0.17  0.24 | | 0.69  0.74 | 0.06 | -0.03 | | 0.15 |
| **Average risk** | **IDM-DE_3_** | 1.44 | 1.17 | | 1.72 |  |  |  | |
|  | **Adj-TA_3_** | 1.54 | 1.27 | | 1.82 | 0.10 | 0.01 | 0.19 | |
| **Higher risk** | **IDM-DE_4_** | 1.55 | 1.28 | | 1.83 |  |  |  | |
|  | **Adj-TA_4_** | 1.76 | 1.49 | | 2.03 | 0.21 | 0.12 | 0.29 | |
| **Highest risk** | **IDM-DE_5_** | 2.36 | 2.08 | | 2.64 |  |  |  | |
|  | **Adj-TA_5_** | 2.69 | 2.40 | | 2.98 | 0.33 | 0.23 | 0.47 | |

Abbreviations: PGS-BMI = Polygenic score – Body Mass Index, IDM-DE = Interventional Disparity Measure – Direct Effect, Adj-TA = Adjusted total association

Supplement Table 4b. Interventional Disparity Measure – Direct Effect (IDM-DE) and adjusted total association (Adj-TA) of categorical PGS-BMI versus the reference category of lowest genetic risk using Avon Longitudinal Study of Parents and Children: estimates and 95% Confidence intervals data, n= 3,347

| **PGS-BMI** | **Estimate** | | | **95% CIs** | | **Difference** | **95% CIs** | |
| --- | --- | --- | --- | --- | --- | --- | --- | --- |
| **Lowest risk (j=1)** | **Reference** | | | | | | | |
| **Low risk (j=2)** | **IDM-DE_2_**  **Adj-TA_2_** | 1.00  1.03 | 0.78  0.81 | | 1.23  1.24 | 0.02 | -0.08 | 0.13 |
| **Average risk (j=3)** | **IDM-DE_3_** | 1.52 | 1.30 | | 1.74 |  |  |  |
|  | **Adj-TA_3_** | 1.71 | 1.48 | | 1.93 | 0.18 | 0.08 | 0.29 |
| **Higher risk (j=4)** | **IDM-DE_4_** | 2.05 | 1.81 | | 2.28 |  |  |  |
|  | **Adj-TA_4_** | 2.35 | 2.10 | | 2.59 | 0.30 | 0.19 | 0.41 |
| **Highest risk (j=5)** | **IDM-DE_5_** | 2.90 | 2.66 | | 3.14 |  |  |  |
|  | **Adj-TA_5_** | 3.34 | 3.09 | | 3.59 | 0.44 | 0.32 | 0.56 |

Abbreviations: PGS-BMI = Polygenic score – Body Mass Index, IDM-DE = Interventional Disparity Measure – Direct Effect, Adj-TA = Adjusted total association

Supplement Table 4c. Interventional Disparity Measure – Direct Effect (IDM-DE) and adjusted total association (Adj-TA) of categorical PGS-BMI versus the reference category of lowest genetic risk using the millennium Cohort Study: estimates and 95% Confidence intervals data, imputed dataset^1^ n= 6,172

| **PGS-BMI** | **Estimate** | | | **95% CIs** | | **Difference** | **95% CIs** | |
| --- | --- | --- | --- | --- | --- | --- | --- | --- |
| **Lowest risk (j=1)** | **Reference** | | | | | | | |
| **Low risk (j=2)** | **IDM-DE_2_**  **Adj-TA_2_** | 0.52  0.60 | 0.35  0.43 | | 0.70  0.77 | 0.08 | 0.02 | 0.13 |
| **Average risk (j=3)** | **IDM-DE_3_** | 1.24 | 1.06 | | 1.43 |  |  |  |
|  | **Adj-TA_3_** | 1.34 | 1.17 | | 1.52 | 0.10 | 0.04 | 0.16 |
| **Higher risk (j=4)** | **IDM-DE_4_** | 1.74 | 1.55 | | 1.92 |  |  |  |
|  | **Adj-TA_4_** | 1.95 | 1.76 | | 2.13 | 0.21 | 0.15 | 0.28 |
| **Highest risk (j=5)** | **IDM-DE_5_** | 2.65 | 2.45 | | 2.84 |  |  |  |
|  | **Adj-TA_5_** | 2.99 | 2.79 | | 3.19 | 0.34 | 0.26 | 0.42 |

Abbreviations: PGS-BMI = Polygenic score – Body Mass Index, IDM-DE = Interventional Disparity Measure – Direct Effect, Adj-TA = Adjusted total association

^1^ Single stochastic imputation by chained equations with 10 burn-in iterations was performed

before the 1,000-fold data expansion. Each of the models used in the imputation was an

expanded version of the models used for estimation.

Supplement Table 4d. Interventional Disparity Measure – Direct Effect (IDM-DE) and adjusted total association (Adj-TA) of categorical PGS-BMI versus the reference category of lowest genetic risk using Avon Longitudinal Study of Parents and Children: estimates and 95% Confidence intervals data, imputed dataset^1^ n= 6,035

| **PGS-BMI** | **Estimate** | | | **95% CIs** | | **Difference** | **95% CIs** | |
| --- | --- | --- | --- | --- | --- | --- | --- | --- |
| **Lowest risk (j=1)** | **Reference** | | | | | | | |
| **Low risk (j=2)** | **IDM-DE_2_**  **Adj-TA_2_** | 0.91  1.01 | 0.73  0.86 | | 1.08  1.17 | 0.11 | 0.02 | 0.20 |
| **Average risk (j=3)** | **IDM-DE_3_** | 1.58 | 1.40 | | 1.76 |  |  |  |
|  | **Adj-TA_3_** | 1.86 | 1.69 | | 2.03 | 0.28 | 0.19 | 0.38 |
| **Higher risk (j=4)** | **IDM-DE_4_** | 1.99 | 1.82 | | 2.17 |  |  |  |
|  | **Adj-TA_4_** | 2.35 | 2.17 | | 2.52 | 0.35 | 0.25 | 0.46 |
| **Highest risk (j=5)** | **IDM-DE_5_** | 3.04 | 2.85 | | 3.23 |  |  |  |
|  | **Adj-TA_5_** | 3.69 | 3.50 | | 3.89 | 0.65 | 0.52 | 0.79 |

Abbreviations: PGS-BMI = Polygenic score – Body Mass Index, IDM-DE = Interventional Disparity Measure – Direct Effect, Adj-TA = Adjusted total association

^1^Single stochastic imputation by chained equations with 10 burn-in iterations was performed

before the 1,000-fold data expansion. Each of the models used in the imputation was an

expanded version of the models used for estimation.
